# Intra-abdominal pressure: an integrative review

**DOI:** 10.1590/S1679-45082016RW3088

**Published:** 2016

**Authors:** Rafaela Milanesi, Rita Catalina Aquino Caregnato

**Affiliations:** 1 Hospital Nossa Senhora da Conceição, Porto Alegre, RS, Brazil; 2 Universidade Luterana do Brasil, Canoas, RS, Brazil

**Keywords:** Intra-abdominal hypertension, Monitoring, physiologic, Nursing care, Intensive care

## Abstract

There is a growing request for measuring intra-abdominal pressure in critically ill patients with acute abdominal pain to be clarified. Summarizing the research results on measurement of vesical intra-abdominal pressure and analyzing the level of evidence were the purposes of this integrative literature review, carried out based on the databases LILACS, MEDLINE and PubMed, from 2005 to July 2012. Twenty articles were identified, in that, 12 literature reviews, 4 descriptive and exploratory studies, 2 expert opinions, one prospective cohort study and one was an experience report. The vesical intra-abdominal pressure measurement was considered gold standard. There are variations in the technique however, but some common points were identified: complete supine position, in absence of abdominal contracture, in the end of expiration and expressed in mmHg. Most research results indicate keeping the transducer zeroed at the level of the mid-axillary line at the iliac crest level, and instill 25mL of sterile saline. Strong evidence must be developed.

## RESUMO

Em pacientes críticos com quadros abdominais agudos a esclarecer é crescente a solicitação da aferição da pressão intra-abdominal. Sintetizar resultados de pesquisas sobre a mensuração da pressão intra-abdominal pela via vesical e analisar o nível de evidência foram os objetivos desta revisão integrativa da literatura, realizada nas bases LILACS, MEDLINE e PubMed, no período de 2005 a julho de 2012. Identificaram-se 20 artigos, sendo 12 revisões de literatura, 4 estudos exploratório-descritivos, 2 opiniões de especialistas, 1 estudo de coorte prospectivo e 1 relato de experiência. O método vesical para mensuração da pressão intra-abdominal foi considerado padrão-ouro. Existem variações na técnica, entretanto pontos em comum foram identificados: posição supina completa, na ausência de contratura abdominal, ao final da expiração e expressa em mmHg. A maioria indica posicionar o ponto zero do transdutor na linha axilar média, ao nível da crista ilíaca e instilar 25ml de solução salina estéril. Evidências fortes precisam ser desenvolvidas.

## INTRODUCTION

The incidence of complications arising from intra-abdominal pressure (IAP) variation^(1–5)^ in critically ill patients suffering from acute abdomen conditions with unconfirmed diagnosis is high and justifies increasing IAP measurement requests.^(1)^

Intra-abdominal pressure is defined as the steady-state pressure concealed within the abdominal cavity and resulting from the interaction between the abdominal wall and viscera; IAP oscillates according to respiratory phase and abdominal wall resistance.^(1–7)^ Intra-abdominal pressure levels up to 5mmHg are considered physiological in adults; however, in patients suffering from conditions devoid of pathophysiological significance, such as obesity, IAP may range from 10 to 15mmHg, while IAP values between 5 and 7mmHg are expected in critically ill patients.^(1–14)^

Intra-abdominal hypertension (IAH) is defined by IAP elevation above 12mmHg in three consecutive measurements taken at 4-to-6-hour intervals. Intra-abdominal pressure may gradually progress to abdominal compartmental syndrome (ACS), with sustained IAP above 20mmHg and associated organ dysfunction or failure.^(1,4–20)^

Abdominal compliance is limited; therefore, high, non-physiological pressure levels interfere with tissue perfusion, potentially leading to severe ischemic or circulatory changes. Correlations between organ dysfunction and increased IAP have been demonstrated in patients suffering from abdominal lesions or conditions, with resulting deterioration of patient's general condition.^(1–4,8,9,11–13,18,19)^

Intra-abdominal pressure measurement system deployment and use are procedures performed by the nursing team; a theoretical background is therefore required if nurses are to properly execute the procedure, which in turn allows early recognition of the problem and ensures medical assistance with lower complication indices.^(6)^ Given IAP measurement reliability is a key factor in the therapeutic decision making process, trained professionals are required for safe application of the technique.^(21)^

However, procedural mismatches in IAP monitoring are evident^(21,22)^ and the correct measurement method remains to be determined, along with proper interpretation of results and establishment of time intervals between measurements.^(1,4,22)^ Scientific, evidence-based analysis is thus required to clarify existing uncertainties and offer quality care to patients. This study was aimed to summarize research findings concerning intravesical IAP measurement in adult patients and analyzing the respective levels of evidence. It is expected that the data presented will contribute to understand IAP and help support the clinical decision making process.

## METHODS

This integrative review comprised six phases: problem delineation, literature search, data collection, critical analysis of selected studies, result presentation and discussion. In the first phase, a core question was formulated: What recent publications are there on intravesical IAP measurement in adults?

In the second phase, databases, literature search strategies, as well as inclusion and exclusion criteria were defined; selected databases were PubMed; MEDLINE and LILACS. Literature searches were based on the descriptor “intra-abdominal hypertension”, selected from the controlled vocabulary list *Descritores em Ciências da Saúde* (DeCS) [Health Science Descriptors]; additional, non-controlled descriptors were selected based on terms that came up frequently in the articles studied during the research project formulation phase, as follows: “intra-abdominal pressure”, “abdominal compartment syndrome”, “acute compartment syndrome”, “compartment syndrome of the abdome”; terms in English, Portuguese and Spanish were considered.

Data collection was performed between June and July 2012; titles and abstracts were investigated for selection of articles meeting the following inclusion criteria: articles published in English, Portuguese or Spanish as of 2005; open access publications; articles discussing intravesical IAP monitoring in adults, particularly those focusing on methodological approaches, fluid instillation volumes and measurement frequency. Articles that did not meet the inclusion criteria proposed, or involved animal models, were excluded.

In the fourth phase, selected articles were analyzed and categorized according to five levels of evidence described in the classification from the Oxford Centre for Evidence-Based Medicine 2011 Levels of Evidence.^(23)^

In the fifth phase, variables extracted from articles were summarized, compared and discussed; result presentation charts were prepared, then submitted to descriptive analysis and confronted with literature data. Results were taken into account in the preparation of this article and organized to contribute to the understanding of the core topic of this review.

## RESULTS

Among potential references resulting from database searches, 20 articles met the inclusion criteria proposed in this study.

As regards publication sources, two articles (10%) were published in Brazilian journals (*Revista Brasileira de Clínica Médica* and *Revista Brasileira de Terapia Intensiva*) and 18 (90%) in the following international journals: *Revista Chilena de Cirugía,* F1000 Reports Medicine, Critical Care Journal, *Cirugía Española,* Critical Care Nurse Journal, Scandinavian Journal of Surgery, Journal of Zhejiang Univerty SCIENCE B, British Journal of Anaesthesia, Acta Clinica Belgica, Critical Care Research and Practice, *Medicina intensiva/ Sociedad Española de Medicina Intensiva y Unidades Coronarias*, Journal of Emergencies Trauma and Shock, Scandinavian Journal of Trauma Resuscitation and Emergency Medicine, and Minerva Anestesiologica.

Most studies were originally from Belgium (7; 35%), United States (3; 15%), Brazil (2; 10%) or Spain (2; 10%); Canada, China, Greece, Italy, United Kingdom and Chile contributed with 30% of the articles (one per country). Therefore, 75% (15) of the studies were published in English, 15% (3) in Spanish and 10% (2) in Portuguese. Publication year distribution was almost linear over the period considered.

Study designs were as follows: literature reviews (60%; 12), exploratory-descriptive studies (20%; 4), specialist's opinions (10%; 2), prospective cohort studies (5%; 1) and experience reports. One study had evidence level 3 and 19 articles had evidence level 5.

Detailed reading permitted further categorization according to article content, as follows: articles discussing intensivists knowledge of IAP, articles focused on ideal saline solution (SS) instillation volume for IAP determination and articles discussing general aspects of intravesical IAP measurement. A summary of articles included in this integrative review is given in charts 1 to 3.

**Chart 1:** Summary of articles focusing on intensivists’ knowledge of intra-abdominal pressure

Objetives	Features	Results
To investigate IAP and IAH knowledge, recognition and management in Italian ICUs^(24)^	Type: exploratory-descriptive study	IAP was measured in 51 ICUs. Lack of IAP measurement was due to lack of a specific kit or unawareness of the technique. The intravesical method was the only one employed. Frequency of serial measurements: every 4 hours in the presence of risk factors (64.7%), or in emergency surgical procedures (21.5%)
	Place: Italy	
	Sample: 77 physicians in charge of ICUs	
	Instrument: questionnaire comprising 9 close-ended questions	
To assess knowledge of ACS, clinical application of IAP measurement, measurement methods and frequency, and criteria for decompressive laparotomy^(25)^	Type: exploratory-descriptive study	IAP was measured by 104 interviewees in suspected cases of IAH/ACS (93.9%). The intravesical method was the only one employed. Frequency: 44.2% in the presence of clinical suspicion; 26.9% every 4 to 8 hours; 15.4% every zero to 4 hours; 10.9% every 12 hours; 2.9% once every 24 hours
	Place: United Kingdom	
	Sample: 137 physicians in charge of ICUs	
	Sample: 137 physicians in charge of ICUs	
To assess physician's knowledge of ACS and respective management characteristics^(15)^	Type: exploratory-descriptive study	IAP was measured in patients clinically predisposed to ACS (51.4%) using the intravesical method (97%), with instillation of 60-100mL of fluid (54.3%), at 4-to-8-hour intervals (60%). Methodological issues were among the major doubts reported in questionnaires
	Place: Brazil	
	Sample: 90 physicians working at 10 ICUs in	
	Rio de Janeiro	
	Instrument: questionnaire comprising 12 close-ended questions	
To determine the current level of understanding and clinical management of IAH/ACS among intensivists working in Chinese hospitals^(16)^	Type: exploratory-descriptive study	IAP was measured by 75 physicians in suspected cases of IAH (88%); the intravesical method was used (100%) with patients in the supine position (97.3%) and using 50-100 mL of fluid (46.7%); the pubic symphysis was taken as the zero-reference point (68%). Out of 33 physicians not measuring IAP, 36.4% were not able to interpret results and 27.3% had never admitted patients suffering from IAH
	Place: China	
	Sample: 108 physicians	
	Instrument: questionnaire comprising 20 close-ended questions	

IAP: intra-abdominal pressure; IAH: intra-abdominal hypertension; ICU: intensive care unit; ACS: abdominal compartment syndrome.

**Chart 2:** Summary of articles focusing on ideal saline solution instillation volume for intra-abdominal pressure measurement

Objetives	Features	Results
To assess the effect of different saline instillation volumes during intravesical pressure measurement^(26)^	Type: prospective cohort study	The intravesical method is the gold standard for indirect measurement. Volumes described ranged from 50 to 300mL. Authors concluded: >50mL may overestimate true IAP; 25mL may be enough; vesical compliance varies within and between patients; a uniform, standardized, accurate and reproducible method is required for multicenter studies
	Place: Belgium	
	Sample: 13 sedated patients submitted to mechanical ventilation	
To describe the significance of saline infusion volume standardization in IAP monitoring^(27)^	Type: specialist's opinion	The intravesical method is the standard technique for indirect measurement, but there's little standardization in literature. The most accurate volume is not clear. Small volumes tend to be employed, as discussed in the studies by Malbrain et al.^(26)^ (25mL) and Waele et al.^(9)^ (10mL), given IAP overestimation is directly proportional to larger volumes.
	Place: Canada	

IAP: intra-abdominal pressure.

**Chart 3:** Summary of articles discussing general aspects of intravesical intra-abdominal pressure (IAP) measurement

Objetives	Features	Results
To provide clinical update for accurate ACS diagnosis and for adequate management and intervention, with particular emphasis on intensive care^(5)^	Type: literature review	Simple, low cost; measurements in mmHg, at end-expiration, supine position, absence of abdominal contractions. Instillation of 25mL, transducer zeroed at mid-axillary line level and connected to 3-way stopcock inserted between vesical catheter and drainage bag. Measured in the presence of two or more risk factors every 4-6 hours; every hour in severe organ dysfunction. Discontinued in absence of acute organ dysfunction or if IAP <10mmHg for 24-48 hours
	Place: Brazil	
To provide updated information, discuss organ dysfunction mechanisms, technique, therapeutic recommendations and treatment^(7)^	Type: literature review	Reference standard for intermittent measurement. Volume: 20-25mL. Expressed in mmHg, measured at end-expiration, in complete supine position, in the absence of abdominal muscle contractions, with transducer zeroed at mid-axillary line level. Recent studies investigated effects of different zero-reference points and elevated head position
	Place: Belgium	
To give a broad overview of IAH/ACS, the role of nurses in assessment, monitoring and collaborative management^(8)^	Type: literature review.	Physical examination not sensitive for IAH detection. Intravesical method is the gold standard for indirect measurement, despite variations; technique adopted is: 20-25mL instillation volume, supine position, zero-reference point at pubic symphysis level, every 4-6 hours in patients at risk, until underlying cause resolution is obtained and IAP ≤12mmHg for 24-48 hours
	Place: United States	
To serve as reference for recommendations defined at the III World ACS Conference^(9)^	Type: experience report	Twelve consensus definitions. About the technique: reference standard for intermittent measurement; in mmHg, at end-expiration, in complete supine position, in the absence of abdominal muscle contractions, transducer zeroed on the mid-axillary line, at iliac crest level, 25mL maximum saline solution instillation volume
	Place: Belgium	
To describe diagnostic criteria, risk populations, monitoring techniques and IAH/ACS management^(10)^	Type: literature review	Gold standard due to reliability, simplicity and low invasiveness. Measured at end-expiration, zeroed at phlebostatic axis level, instillation of 25-50mL. Transient increase in sedation suggested to reduce interferences. Potential routine protocol: every 2 hours for the first 8 hours following ICU admission; every 4 hours over the next 8 hours; and every 8 hours over the following 24 hours
	Place: United States	
To review risk factors for IAH/ACS, related conditions, pathophysiology, diagnostic methods and therapeutic advancements^(11)^	Type: literature review	Physical examination and diagnosis have low sensitivity for IAH detection. Intravesical method simple, reliable, reproducible, minimally invasive, low cost, measured at end-expiration, in supine position, zero-reference point on the mid-axillary line at iliac crest level, in the absence of abdominal contractions, instillation of 25mL saline solution
	Place: Spain	
To provide a general overview and present historical aspects, definitions, pathophysiology and suggestions for IAH/ACS management^(12)^	Type: literature review	Physical examination and imaging modalities not sensitive for IAH detection. Safe, efficient monitoring: ≥2 risk factors, one baseline measurement; if IAH, serial measurements. Intravesical method is simple, low cost, thought to be the gold standard. Measurements taken with patient in supine position
	Place: Greece	
To present currently accepted consensus definitions regarding IAH and ACS diagnosis and treatment^(13)^	Type: literature review	Clinical assessment has low sensitivity in detection of increased IAP. Intravesical method more widely employed due to simplicity, low cost and minimal risk. Expressed in mmHg (=1.36cmH_2_O), at end-expiration, in complete supine position, in the absence of abdominal muscle contractions, zero-reference point at mid-axillary line level, instillation volume ≤25mL
	Place: Belgium	
To discuss etiology, epidemiological data, measurement techniques, diagnosis, complications, prevention	Type: literature review	Worldwide acceptance due to simplicity and minimal cost, but technique varies. Measured in mmHg, at end-expiration, in complete supine position, in the absence of abdominal contractions, with transducer zeroed at mid-axillary line level and maximal saline solution instillation volume of 25mL and treatment^(14)^
	Place: Belgium	
Not mentioned^(17)^	Type: literature review	Clinical assessment inaccurate for IAH detection. Measure: upon admission of critically-ill patient, in the presence of risk factors or clinical deterioration. Other hollow organs were described, but none as simple and user-friendly as the bladder. Patient in supine position, pressure module zeroed on the mid-axillary line at iliac crest level
	Place: United States	
To discuss etiology, epidemiological data, IAP measurement, diagnosis, complications, prevention and treatment options for ACS^(18)^	Type: literature review	Physical examination and imaging modalities inaccurate for diagnosis but indicate causes. IAP measured at one site is assumed to reflect IAP as a whole. Intravesical method is the gold standard due to simplicity and minimal cost. Several tools have been developed, such as Foley Manometer or AbViser stopcock. Continuous technique has been described, but is not widely used
	Place: Belgium	
Not mentioned^(19)^	Type: literature review	Patients must be in complete supine position and abdominal muscle contraction must be absent; transducer is zeroed at pubic symphysis or mid-axillary line level. Instillation of 25mL of sterile saline solution; values obtained must be expressed in mmHg
	Place: Spain	
Not mentioned^(20)^	Type: literature review	Clinical assessment has low sensitivity (40%) for IAH estimation. IAP increasingly employed due to relevance of early IAH detection and management; transducer zeroed at pubis level. Original technique employed 50-100mL but recent study reported better correlation with IAP when 50mL are used
	Place: Chile	
Not mentioned^(28)^	Type: specialist's opinion	Despite increased attention given to topic, general clinical application has not been established to date. Current consensus on ideal measurement method or time point is lacking. Future research efforts should aim to improve consensus definitions concerning IAH and ACS
	Place: Belgium	

ACS: abdominal compartment syndrome; IAH: intra-abdominal hypertension; IAP: intra-abdominal pressure; ICU: intensive care unit.

## DISCUSSION

Organ perfusion compromise may develop whenever intracompartmental pressure exceeds capillary blood pressure. Deleterious consequences of IAP have been described more than 150 years ago; still only over the two last decades have IAP-related concerns been rediscovered and better characterized, with recently acquired clinical significance.^(5,13,15,25)^

Historically, several authors have tried to come up with an ideal IAP measurement method. Using a tube connected to a manometer, Schatz was able to measure intrauterine pressure in 1872. One year later, Wendt measured IPA through the rectum and, in 1875, Odebrecht did the same in the urinary bladder.^(12)^ The IAH concept developed by these researchers was put aside after the World War I and rediscovered by the end of the 20^th^ century, through the pioneer work of Kron, Harman and Nolan (1984),^(5,8,12,13)^ describing the original IAP measurement method. Bearing in mind the ability of the urinary bladder to act as a passive container at volumes of 50 to 100ml, these authors hypothesized IAP could be accurately measured via an indwelling urinary catheter (IUC).^(29)^

Increased IAP is common in critically ill patients and is thought to be an independent mortality predictor.^(8,10,13,16,18,25,26)^ A prospective study involving 13 intensive care unit (ICUs) in Belgium, Austria, Israel, Brazil and Australia reported IAH and ACS prevalence of 32.1% and 4.2%, respectively, upon patient admission.^(5,16)^ A second study revealed significantly higher mortality indices in critically ill patients presenting with increased IAP compared to unaffected patients (37.9% and 19.1% respectively).^(8,16)^ These data suggest increased IAP is common among critically ill patients, and may increase morbidity and mortality risks.

However, IAP measurement is not recommended for all ICU patients. The major indications comprise patients presenting with two or more risk factors, patient screening upon ICU admission or presence of progressive or new organ failure.^(5,12,16)^ In such cases, IAP should be monitored at 4-to-6-hour intervals, with hourly IAP monitoring restricted to patients suffering from severe organ dysfunction.^(5,8,12,17)^ In actual fact, in many cases IAP measurement is restricted to suspected cases of IAH;^(15)^ hence, implementation of routine IAP monitoring is paramount for IAH/ACS recognition and effective treatment.^(10,13)^

Massive fluid replacement, polytransfusion, open abdomen management, hypothermia, coagulopathy, systemic inflammatory response syndrome, severe sepsis or septic shock, hepatic dysfunction with ascites, mechanical ventilation and positive-end expiratory pressures (PEEP) above 10cmH_2_O are the major risk factors for IAH and ACS.^(5,10)^

Although IAH and ACS are not synonymous, both conditions reflect different stages of the same pathological process^(5)^ and affect all organ systems, with more evident manifestations in ACS.^(16,26,27)^ Clinical presentation generally includes a tense, distended abdomen, hypotension, high airway pressure, hypercapnia and oliguria.^(25)^

Clinical examination is thought to be a poor, low sensitivity (40 to 60%) IAP estimation tool.^(8,11–13,17,18,20,27)^ Abdominal circumference is another low sensitivity IAP estimation method, as are available imaging techniques, which basically provide etiological data and help support decision making.^(8,11–13,17,18,20,27)^ Therefore, multidisciplinary team members must be aware of proper IAP measurement techniques for correct diagnosis and management of IAH patients.^(8,12,26)^ Inaccurate IAP measurements or failure to convert values given in cmH_2_O into mmHg may translate into incorrect indications for surgical abdominal decompression, with increased complication risks for patients. Also, erroneous recognition of IAH as the cause of the patient's condition may lead to changes in support treatment and ventilation parameters, or mislead physicians into ruling out other diagnostic possibilities.^(27)^

Early recognition and proper staging of risk patients are vital for effective treatment. The management is based on four principles: (1) serial IAP monitoring; (2) systemic organ perfusion optimization; (3) introduction of specific procedures aimed at IAP control and mitigation of potential consequences for target organs; and (4) fast surgical decompression in refractory ACS cases. Three algorithms (assessment, management and medical management) can be found at the World Society of the Abdominal Compartment Syndrome (WSACS) website at www.wsacs.org.^(16,27)^

Despite the growing number of publications over the last few years, IAP measurement techniques and clinical applicability have not been fully established to date.^(15,16,25,28)^ As clearly demonstrated in studies involving intensivists (Chart 1). Intravesical IAP measurement is a consensus; however, zero-reference point, infusion volume, measurement frequency and indications remain debatable.^(15,16,24,25)^

Lack of consensus definitions and the confusion arising from constraints related to interstudy comparisons fostered the creation of the WSACS, a non-for-profit society founded by a multinational group of physicians, in 2004, with the purpose of promoting research and education, as well as improving survival of IAH/ACS patients.

The first WSACS conference, held in 2004, represented a milestone with respect to normalization of definitions, diagnostic criteria, treatment modalities and recommendations concerning future research topics, always based on robust evidences, recommendations and the 12 consensus definitions. As to IAP measurement techniques, definitions 4 and 5 should be emphasized. One definition states that IAP should be expressed in mmHg (1mmHg equals 1.36cmH_2_O) and measurements performed at end-expiration, with the patient in complete supine position and in the absence of abdominal muscle contractions; the transducer should be zeroed on the mid-axillary line, at the level of the iliac crest. According to another definition, the reference standard for intermittent IAP measurement is via the bladder, with a maximal infusion volume of 25mL of sterile saline.^(13,16,18,19)^

The WSACS lists laparoscopic and intravesical IAP measurement as the direct and indirect measurement methods of choice respectively.^(5,8,10,18,26,27)^ However, regardless of wide intravesical IAP measurement acceptance, instillation volumes have not been standardized and may range from 50 to 300ml.^(10,18,26)^ In one study testing different instillation volumes (Chart 2), high volumes were associated with high IAP values; overestimation was thought to result from increased intrinsic pressure, in contrast with data given by Kron et al.^(29)^ Pressure elevation was already relevant at the 25mL level, but acquired statistical significance from 75mL in most patients. Given the lack of robust data, the authors of that study suggested maximal instillation volumes of 25mL would be enough to create a fluid column and remove air. These results were supported by De Waele et al.^(9)^ who considered 10mL to be enough for IAP estimation. The same study also pointed out that fluid temperature, as well as infusion speed and volume, may lead to contraction of the detrusor muscle of the bladder; slower infusion of warm (body temperature) fluid is therefore recommended, with measurements taken 30 to 60 seconds after Instillation.^(26,27)^

Intravesical IAP measurement is currently the most widely accepted technique given its simplicity,^(5,10–14,17,18)^ reliability,^(10,11)^ user-friendliness^(1,7)^ and reproducibility,^(11,13)^ with the added benefits of low cost,^(5,11–14,18,20)^ little invasiveness^(10,11)^ and minimal complication risks.^(13)^

The original description given by Kron et al. is as follows: using an IUC and with the closed-system urine drainage bag tubing clamped, the bladder is instilled with 50 to 100mL of saline solution; a pressure transducer or water column is then connected via a 16-gauge needle inserted through the aspiration port located at the proximal portion of the extensor tube for IAP estimation. The transducer should be zeroed at and the fluid column leveled with the pubic symphysis, with the patient in the dorsal position.^(29)^

Variations of the original technique have been described. Currently, tools for IAP measurement are readily available in hospital units and specialized WSACS approved kits can be purchased; kit selection should be based on features such as reproducibility, team safety, efficacy and cost.^(8,18)^

**Figure 1:**
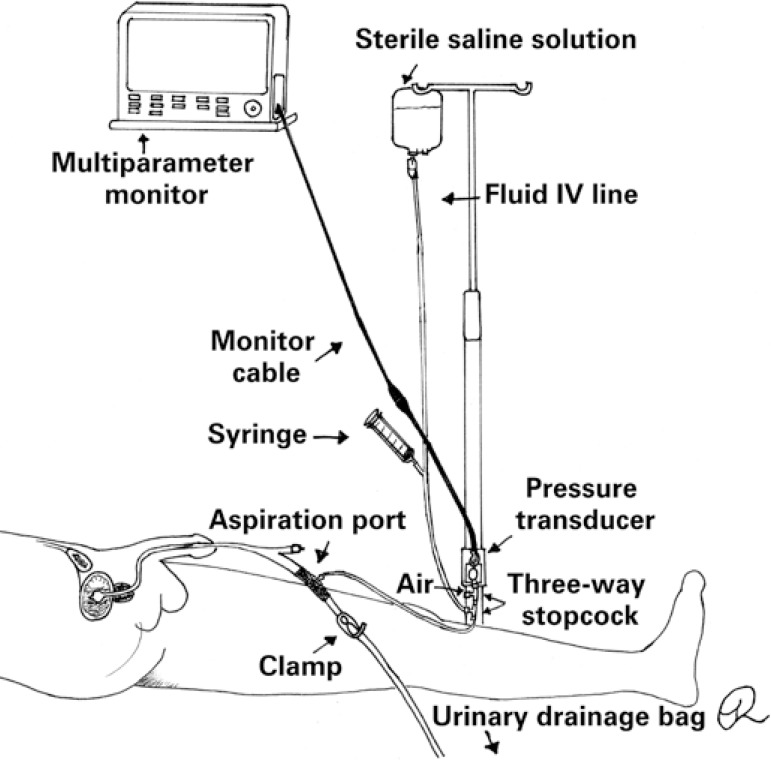
Intra-abdominal pressure measurement using the pressure transducer technique

Intra-abdominal pressure measurement using pressure transducers is shown in figure 1. The assembled system is filled with saline solution and connected to the aspiration port. With the transducer positioned at point zero, the drainage bag tube clamp is closed; aspiration and instillation of 25mL of saline follows and readings are then performed. The drainage bag tube clamp must be released at the end of the procedure.^(8)^

**Figure 2:**
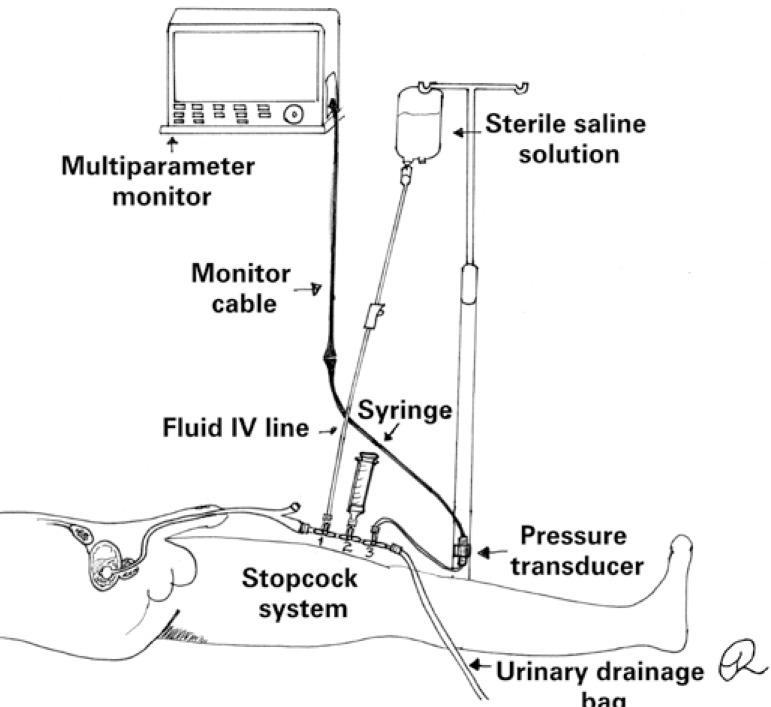
Intra-abdominal pressure measurement using the pressure transducer technique and a three-way stopcock system

Intra-abdominal pressure measurement using a pressure transducer connected to a stopcock system is shown in figure 2. With the system set up and filled with SS, and the transducer positioned at point zero, the first stopcock is turned off to the patient and on to the fluid bag; the stopcock to the syringe is open and saline solution (25mL) is aspirated. The first stopcock is turned off to the fluid bag and, leaving the second stopcock open (from the syringe to the IUC), SS is instilled. The second stopcock is the turned off to the syringe and the third stopcock adjusted so as to interrupt the flow towards the drainage bag. After ensuring the third stopcock is turned off to the transducer, the flow towards the drainage bag is released.^(8)^

**Figure 3:**
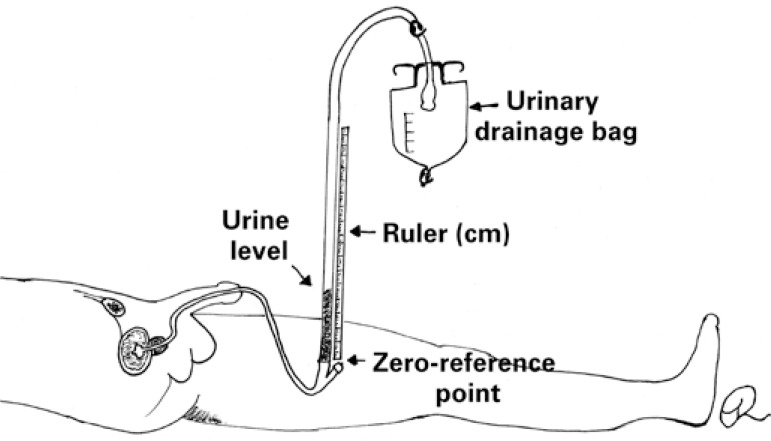
Intra-abdominal pressure measurement using the U-tube technique

Intra-abdominal pressure measurement using the U-tube technique figure 3 lacks robust clinical validation and is recommended for tracking only. A ruler (in centimeters) is placed vertically at point zero; the urinary catheter is then raised and the proximal aspect of the urinary drainage bag leveled with the starting point of the ruler; finally, measurements are taken using the fluid column that is formed.^(8)^

**Figure 4:**
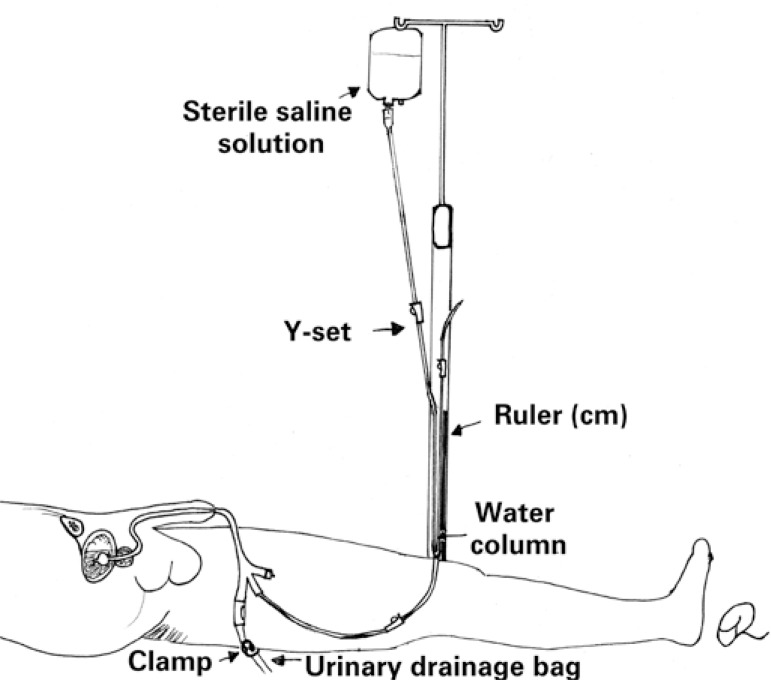
Intra-abdominal pressure measurement using the Y-set technique

A fourth IAP measurement technique figure 4 commonly used in Brazilian hospitals, employs a Y-set. With the fork leveled with the zero point of a measuring tape (cm) and a fluid bag SS connected to one end, both ends are laid over the ruler. The system is then filled with saline and connected to the third IUC stopcock prior to drainage bag tube clamping and infusion of 25mL of SS. Within 30 to 60 seconds the measuring system is opened to the catheter and water column and pressure readings in cmH_2_O taken and converted into mmHg. The measuring system is closed and the urinary drainage bag clamp released at the end of the procedure.^(1,2)^

A continuous measuring technique has been described, but standardization level is low and application and benefits are disputable.^(8,27)^

## CONCLUSION

This study clarified some aspects of intra-abdominal pressure measurement. Methodological variations recognized by the World Society of the Abdominal Compartment Syndrome have been highlighted. Inaccuracy of clinical assessment and imaging modalities in intra-abdominal pressure determination was shown to be a consensus, with intravesical intra-abdominal pressure measurement being the method of choice due to simplicity and low cost.

Technical aspects found support in consensus definitions: Intra-abdominal pressure should be expressed in mmHg and measured in the supine position, at end-expiration and in the absence of abdominal muscle contraction.

With regards to methodological differences, most articles recommended transducer zeroing on the mid-axillary line, at iliac crest level, while three suggested the pubic symphysis as the zero-reference point. Sterile saline instillation volume is variable; however, a maximal volume of 25mL is recommended in most studies. Inherent procedural risks were not discussed.

Doubts regarding intra-abdominal pressure measurement in clinical practice persists despite widely available, normalized information. Studies providing robust evidences are lacking.
